# Keating’s “Cyclopedia of Diseases of Children”

**Published:** 1889-08

**Authors:** 


### Book Notices.

Cyclopædia of the Diseases of Children.—Edited by J. M. Keating, M. D., Vol. 1, illustrated, J. B. Lippincott & Co., 1889, Philadelphia. Pages 981; price $5 per Vol.

Here is something new and good, excellent. It is a departure from the fashion of culling the "best" from various text-books, and compiling it under a new name, and it is going to have a big sale—become popular, and take its place as a standard work, as did Ziemssen's General Cyclopœdia, which it resembles in the fact that it consists of a series of monographs, written by men selected with especial reference to their respective ability, observation and experience in the department assigned them. As the authors says, "the articles were written especially for this work, by American, British and Canadian Authors." The names of Keating, Parvin, J. C. Warren, Jacobi, S. S. Adams, Bartholow, Shakespeare and Matas, will strike the reader as very familiar in connection with researches in Pediatrics, and in fevers generally.

The "preface" says, and we do not believe we could express it better; the work is "arranged in the form of a systematic treatise, and devoted to the consideration of the anatomy, physiology, medicine, surgery and hygiene of infancy, childhood puberty and adolescence;" the object being to "include not only the medicine and surgery of Pediatrics, but also all the specialties tributary to it, as well as all collateral subjects of interest and importance, many of which can not be found treated in any other work of this character." It also includes "articles upon prominent symptoms, such as cough, convulsions etc., the proper significance of which could be estimated only after laborious research, unless the matter were considered in special chapters."

A pretty wide scope, one will say; almost as stupendous in its grasp as the movement now being made to "combine" under one management the sugar interest of the world, or the "trust" to control the manufacture of cotton cloth in America. But judging from the beginning made—the plan will be carried out in a most satisfactory manner.

Dr. Keating has here presented to the profession in an attractive form, the very cream, not of the literature of the subject—but of the subject. The articles are all new and original, and, as said, are written by men who have thought, studied and observed children's diseases at the bedside. All that is known about sick children, is here presented, together with the treatment which has been found most successful by masters in the art.

But the work, notwithstanding its name, is not confined to diseases of children. Some of the writers embrace their subject—typhus fever and dengue for instance, in its entirety, and thus additional value is given the work; it is textbook on fevers.

Where all is good, it would appear unfair to select any one chapter for commendation; but our own colleague and fellow citizen, Dr. J. W. McLaughlin, having been the first to demonstrate and cultivate the micrococus of dengue—thereby throwing new light on the etiology and pathology of that little understood disease, the chapter on Dengue, by Dr. Rudolph Matas of the Charity Hospital staff, New Orleans, naturally attracted our attention, especially as he not only refers to Dr. McLaughlin's original work, but quotes largely from the doctor's writing on the subject, submitting also, the micro-photographs of the cocci, taken from drawings, made by Dr. McLaughlin. He also pays the Doctor the following handsome tribute:

"As to the *vera causa*, the essential ferment or contagium, which is the *sine qua non* in the etiology of the disease, our knowledge was still more deplorably scanty, until the advance of modern bacteriological research gave strength to the numerous suggestions that had been offered by almost every writer on the disease since the earlier glimmering of the panspermist doctrine. All ideas in this direction were entirely conjectural and hypothetical until 1885, when the unusually fertile and rich field offered through the great epidemic, which prevailed in the State of Texas, was utilized by Dr. J. W. McLaughlin, of Austin, and the first steps taken to examine the etiological problem with all the lights that modern science could lend to the inquiry."

"The facts obtained by him were sufficiently striking and valuable, if confirmed by further investigation, to support the most sanguine expectations that may be entertained as to the early and positive discovery of the essential etiological factor." (Here Dr. Matas incorporates in his excellent treatise Dr. McLaughlin's original account of his discovery—and the technique of his operations as published in the *Journal of the American Med. Association*. ("Researches into the Etiology of Dengue.")

Dr. Matas' "differentiation between Yellow fever and Dengue," shows deep research, and familiarity with both diseases—so much alike in their main features as to cause much confusion and alarm. He makes it very clear on paper, but it is most difficult in practice, especially when yellow fever is threatening, and the first "suspicious case" occurs.

The volume closes with a fine chapter on the General Therapeutics of Children's Diseases, by Prof. Bartholow, which is a book within itself, worth the price of the volume. Previous to that is a Chapter on Embryology, by Horace Jayne, M. D., and before that, one on Joined Twins, by Wm. Wright Jaggard, M. D. This chapter contains a complete resume, with illustrations, of all the "double monsters," heretofore reported, which is most interesting.

Messrs. Lippincott & Co., realizing the extraordinary value of the work, have done their share—the mechanical—in most creditable style. The book is a splendid specimen of the book-makers art.

